# The Thanatomicrobiome: A Missing Piece of the Microbial Puzzle of Death

**DOI:** 10.3389/fmicb.2016.00225

**Published:** 2016-02-24

**Authors:** Gulnaz T. Javan, Sheree J. Finley, Zain Abidin, Jennifer G. Mulle

**Affiliations:** ^1^Forensic Science Program, Physical Sciences Department, Alabama State UniversityMontgomery, AL, USA; ^2^Ph.D. Program in Microbiology, Department of Biological Sciences, Alabama State UniversityMontgomery, AL, USA; ^3^Department of Epidemiology, Rollins School of Public Health, Emory UniversityAtlanta, GA, USA; ^4^Department of Human Genetics, School of Medicine, Emory UniversityAtlanta, GA, USA

**Keywords:** thanatomicrobiome, epinecrotic communities, death, microbial forensics, putrefaction

## Abstract

Death is a universal phenomenon; however, is there “life after death?” This topic has been investigated for centuries but still there are gray areas that have yet to be elucidated. Forensic microbiologists are developing new applications to investigate the dynamic and coordinated changes in microbial activity that occur when a human host dies. There is currently a paucity of explorations of the thanatomicrobiome (*thanatos*-, Greek for death) and epinecrotic communities (microbial communities residing in and/or moving on the surface of decomposing remains). Ongoing studies can help clarify the structure and function of these postmortem microbiomes. Human microbiome studies have revealed that 75–90% of cells in the body prior to death are microbial. Upon death, putrefaction occurs and is a complicated process encompassing chemical degradation and autolysis of cells. Decomposition also involves the release of contents of the intestines due to enzymes under the effects of abiotic and biotic factors. These factors likely have predictable effects on postmortem microbial communities and can be leveraged for forensic studies. This mini review provides a critical examination of emerging research relating to thanatomicrobiome and epinecrotic communities, how each is studied, and possible strategies of stochastic processes.

## Introduction

The Human Microbiome Project (HMP) demonstrated that healthy adults contain about 10 trillion human cells, but as many as a debatable 10–100 trillion microbial cells depending on the location (Turnbaugh et al., 2007; Hamady and Knight, 2009; Sender et al., 2016). The HMP also revealed that a healthy adult contains body-site specific microbiomes with an astonishing diversity in and between each microbiome (The Human Microbiome Project Consortium, 2012). Current knowledge of the human postmortem microbiome (i.e., thanatomicrobiome and epinecrotic communities) and their applications have the potential to transform forensic microbiology.

Microbiome research to date has focused on the commensal and pathogenic microbes that contribute to health outcomes, but little is known about the succession of microorganisms after a human host dies. The purpose of this mini review is to highlight (i) the components of the human postmortem microbiome; (ii) the recent progress in molecular and high-throughput technologies that are advancing our knowledge of body-site specific microbiomes of corpses; and (iii) the future directions of the human postmortem microbiome and related studies for the estimation of time of death.

### The Human Postmortem Microbiome

**FIGURE 1:**
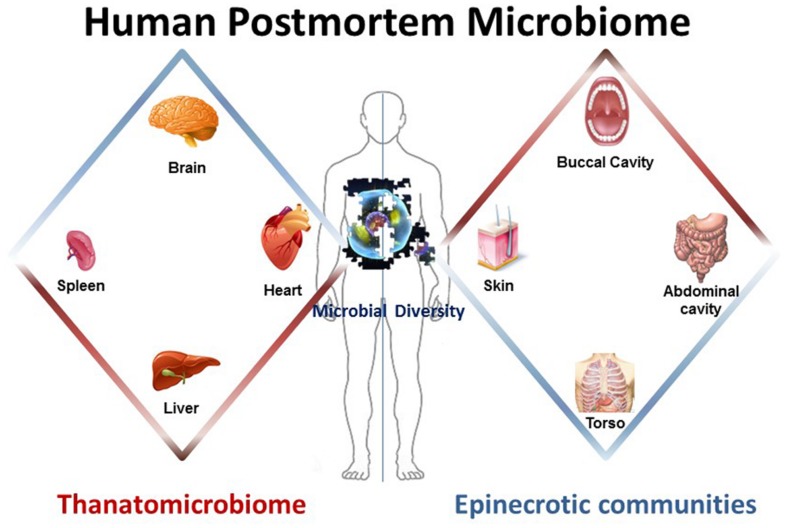
**The human postmortem microbiome.** The components of the human postmortem microbiome include the thanatomicrobiome (the microbiome of internal organs of cadavers) and epinecrotic microbial communities (the microbiome on surfaces of decaying remains).

Components of the human postmortem microbiome include the thanatomicrobiome (Hyde et al., 2013, 2015; Tuomisto et al., 2013; Can et al., 2014; Damann et al., 2015; Hauther et al., 2015) and epinecrotic microbial communities (Dickson et al., 2011; Pechal et al., 2013, 2014; Benbow et al., 2015; Metcalf et al., 2016), (**Figure 1**) (**Table 1**), and each has the potential to provide a missing piece of the postmortem microbial puzzle. Human postmortem microbiome studies have demonstrated that these communities are present in the host antemortem or colonize on the body after a human or animal surrogate dies. Presently, there is a paucity of studies that investigate the human thanatomicrobiome (“microbiome of death”) and epinecrotic communities (microbial communities residing in and/or moving on the surface of decomposing remains).

**Table 1:** Select postmortem microbiome studies using humans and animal surrogates.

Cases	Specimen(s)	Detection/Sequencing platform(s)	Microbiological finding(s)	References
**Thanatomicrobiome studies**				
12 Humans	Proximal large intestine	RT-qPCR	*Bacteroides* and *Lactobacillus* can be used as quantitative markers of PMI	Hauther et al., 2015
12 Humans	Lower rib	Roche GS-FLX Titanium pyrosequencing	99.2% of sequences were from 6 bacterial phyla: Proteobacteria, Firmicutes, Bacteroidetes, Actinobacteria, Acidobacteria, and Chloroflexi	Damann et al., 2015
11 Humans	Brain, heart, liver, spleen, and blood	Roche 454-FLX-Titanium pyrosequencing	Anaerobic *Clostridium* sp. and *Lactobacillus sp.* were the predominant bacteria	Can et al., 2014
2 Humans	Mouth, GI tract, and general body cavity (2013); Mouth, skin, and rectum (2015)	Roche 454-FLX-Titanium pyrosequencing	A significant percentage of Firmicutes, Bacteriodetes, and Actinobacteria were detected. A marked shift from aerobic to anaerobic bacteria in all tissues	Hyde et al., 2013, 2015
33 Humans	Blood, liver, portal vein, mesenteric lymph node, and pericardial fluid	Bacterial culturing and RT-qPCR	21 bacteria genera were detected. The five most abundant species were: *Staphylococcus* sp., *Streptococcus* sp., *Clostridium* sp., *Enterococcus* sp., and *Escherichia* sp.	Tuomisto et al., 2013
**Epinecrotic microbial communities**				
6 Swine	Buccal cavity, skin, and interior anal cavity	Roche 454-FLX-Titanium pyrosequencing	Developed a framework to explain 94.4% of PMIs to within 3 h of actual PMIs	Pechal et al., 2014
3 Swine	Buccal cavity and skin	Roche 454-FLX-Titanium pyrosequencing	Proteobacteria and Firmicutes were the predominate phyla. A shift from Proteobacteria to Firmicutes in later decomposition stages	Pechal et al., 2013
3 Swine Heads (marine submersion)	Cheek skin, snout and neck	ABI capillary electrophoresis sequencing	Identified 15 orders, 21 families, and 39 genera of bacteria. Seasonal successional patterns were observed	Dickson et al., 2011

*The cases, examined specimen(s), detection/sequencing platform(s), and microbiological finding(s) for selected postmortem microbiome studies.*

## Thanatomicrobiome

The human thanatomicrobiome is a relatively new term derived from *thanatos*-, which is Greek for death, and it is the study of the microorganisms found in internal organs and cavities upon death (Hyde et al., 2013, 2015; Tuomisto et al., 2013, 2014; Can et al., 2014; Damann et al., 2015; Hauther et al., 2015). The thanatomicrobiome encompasses the complete aggregation of microorganisms (e.g., bacteria and fungi) found in distinct body locations of decomposing corpses. The significant impact of these studies is the potential to provide forensic data to be utilized as microbial physical evidence in medicolegal death investigations.

It is a long held belief that human internal organs are sterile in living hosts due to uncultivable microorganisms (Ford, 1901; Stewart, 2012). However, Fredette (1916) discovered culturable organism(s) in 35% of cases of postmortem anatomical dissection. Interestingly, the ratio of positive cases increased as the postmortem interval (PMI) increased. Therefore, the microbes discovered in the internal organs of cadavers presumably represent those that are directly associated with decomposition. For example, cadaver liver has the highest microbial diversity among all internal organs (Can et al., 2014). This diversity is potentially caused by many factors including (i) the liver is the largest solid organ in the human body, with a mass of 1400–1800 g and all metabolism takes place in this organ; (ii) it receives nutrient-rich blood from two sources, the hepatic portal vein and artery, and its location is in close proximity to other organs that have high microbial content (i.e., gastrointestinal tract); and (iii) pancreatic enzymes, stomach acids, and gallbladder fluids initiate decomposition and create rapid autolysis that spreads immediately to the liver (Sibulesky, 2013). Moreover, the presence of microorganisms in other internal organs arise through antemortem bacteremia, perimortem agonal spread during the process of dying, and postmortem translocation from epinecrotic surfaces into blood and body tissues (Morris et al., 2006).

### Assessing the Methodology of the Thanatomicrobiome

According to the Hyde et al. (2013, 2015) thanatomicrobiome studies, cadaver specimens were collected by sterile swabbing of the study area, and a soil DNA extraction kit was used to isolate bacterial DNA as reported in Caporaso et al. (2011). Alternatively, in the Can et al. (2014) study, criminal cadaver cases were analyzed in two ways: dissection of tissues followed by the phenol/chloroform method of DNA extraction versus swabbing organ tissues and blood followed by the heating/thawing method. The results of both Hyde and Can studies confirmed the efficacy of swabbing methods similar to those perfected by HMP protocols. According to thanatomicrobiome studies, the results revealed that the swabbing method provided a higher microbial diversity. 16S rRNA gene amplicon-based sequencing approaches has made significant progress in opening up new lines of inquiry. These high-throughput techniques have revolutionized the detection of thanatomicrobiomic succession patterns that are not usually detected by traditional, culture-based methods. Also, these methods have made it possible to reconstructing the sequences of large genomic data or the functional processes of a selection of its genes.

### Research Models of the Thanatomicrobiome

Many postmortem microbiological studies use animal models such as swine (Benninger et al., 2008; Carter et al., 2008, 2010, 2015; Howard et al., 2010; Benbow et al., 2015) or juvenile rodents (Carter et al., 2008; Metcalf et al., 2013, 2016). Swine represents an excellent surrogate due to its high degree of similarity during processes of human decomposition (Campobasso et al., 2001). When human cadavers are used to characterize the thanatomicrobiome, they are frequently obtained through field experiments using willed body donation (Hyde et al., 2013, 2015; Damann et al., 2015; Hauther et al., 2015; Metcalf et al., 2016) or autopsied cadavers in criminal cases (Tuomisto et al., 2013, 2014; Can et al., 2014). There are inherent limitations in the use of donated cadavers including the inability to control the PMI and the limited number of replications that are possible with animal studies.

### Substantial Findings of the Thanatomicrobiome

A recent informative thanatomicrobiome investigation by Hauther et al. (2015) set out to quantify the postmortem successional patterns for the bacterial communities of six cadavers decomposing at the University of Texas Anthropological Research Facility (ARF). The proximal large intestines were swabbed at 9–20 days, and real-time quantitative PCR (RT-qPCR) with group-specific primers targeting 16S rRNA genes to probe for three target bacteria commonly found in the gut; namely, *Bacteroidetes, Lactobacillus*, and *Bifidobacterium*. *Bacteroides* and *Lactobacillus* sp. demonstrated repeatable, significant (*p* < 0.05), and exponential reduction in relative abundance as PMI increased. These bacteria are anaerobic, intestinal bacteria that decline in abundance over time due to the accumulation of gasses that are released during autolysis and initiate oxidative stress on anaerobic bacteria (Heimesaat et al., 2012). Therefore, according to Heimesaat et al. (2012), these two bacteria potentially provide a framework for the development of medicolegal quantitative indicators of PMI.

Another major contribution to thanatomicrobiome studies investigated bacterial migration from the gut into the blood, liver, portal vein, mesenteric lymph node (MLN), and pericardial fluid of cadavers using culturing and RT-qPCR techniques (Tuomisto et al., 2013). The results identified five highly abundant species (*Staphylococcus* sp., *Streptococcus* sp., *Clostridium* sp., *Enterococcus* sp., and *Escherichia* sp.), and a total of 21 different bacteria genera. The findings most relevant to thanatomicrobiome studies were that cadaver tissue samples should be sampled within 7 days, and the optimal body sites were liver and pericardial fluids, which remain most microbe-free within 5 days. In a related thanatomicrobiome study, the relative amounts of commensal gut bacteria that translocated into liver and ascites of autopsied cirrhotic livers demonstrated that *Enterobactericaea* were the most common bacteria translocated into diseased hepatocyte tissues (Tuomisto et al., 2014). The significance of this finding is that it confirms previous findings that *Enterobactericaea* are facultative anaerobes that survive on fermenting sugars, which are in high abundance in cirrhotic livers (Williams et al., 2010).

Damann et al. (2015) used high-throughput sequencing methods to determine the diversity of bacterial communities of partially skeletonized lower rib bones of 12 corpses decomposing at ARF. Their results demonstrated that 99.2% of the sequences were from six bacterial phyla: Proteobacteria, Firmicutes, Bacteroidetes, Actinobacteria, Acidobacteria, and Chloroflexi. Furthermore, the communities were similar to bacteria associated with the gut. However, as the bones advanced to the dry skeletal remains stage, the bacterial communities began to resemble soil microbial signatures. From these results, the authors suggested that the obligotrophic nature of corpse bones, in contrast to the copiotrophic nature of soft tissue, and the protection of organic nutrients provided by the bones blocked bacterial blooms in the initial years of skeletonization.

## Epinecrotic Communities

Another major component of the human postmortem microbiome is epinecrotic microbial communities, which are comprised of prokaryotes, protists, fungi and other microeukaryotes residing in and/or moving on the surface of decomposing remains (Pechal et al., 2013, 2014; Benbow et al., 2015). The surfaces include superficial epithelial tissues, oral mucosal membranes, and distal orifices of the alimentary canal. Antemortem studies have demonstrated the potential for bacteria on the exterior of the body to be used as molecular markers for forensic identification. For example, studies of skin-associated bacteria taken from epithelial swabs have shown that microbial communities have a high biodiversity that exhibit distinct variability between individuals (Fierer et al., 2010). Additionally, skin bacteria are highly resistant to environmental factors (i.e., UV, humidity, and temperature). Therefore, microbes identified on epinecrotic tissues are potentially suitable as molecular “fingerprints” for postmortem microbiological studies.

### Assessing the Methodology of Epinecrotic Communities

Epinecrotic studies use either Roche 454-FLX-Titanium pyrosequencing (Pechal et al., 2013, 2014) or ABI capillary electrophoresis sequencing (Dickson et al., 2011). Sampling protocol usually involved non-abrasive swabbing of the surface of decomposing remains with sterile cotton applicators. For example, Pechal et al. (2013, 2014) sampled epinecrotic communities by swabbing 2.54 cm x 15.24 cm sections of two areas (skin and buccal cavity) of swine carcasses, and other researchers swabbed a smaller area of 2 cm × 2 cm of skin (Dickson et al., 2011). Generally, DNA is extracted by bead-beating then isolated with phenol-chloroform protocols (Dickson et al., 2011; Pechal et al., 2014) or extracted using standard protocols as outlined in Caporaso et al. (2011). Culture-dependent methodologies were recently revisited to study the epinecrotic ecology of aerobic bacteria on the skin of head and limbs of decomposing swine (Chun et al., 2015).

### Research Models of Epinecrotic Communities

Epinecrotic research on human corpses most often use cadavers decaying in natural ecosystems. As with thanatomicrobiome studies, there are essential limitations in the number of available cases. For example, two Hyde et al. (2013, 2015) studies examined epinecrotic communities on superficial tissues of only two cases in each study. Other studies have used animal surrogates such as swine (Dickson et al., 2011; Pechal et al., 2013, 2014; Chun et al., 2015) or mice (Metcalf et al., 2013, 2016) which are commonly used to mimic human decomposition. Metcalf et al. (2013, 2016) examined epinecrotic communities, as well as gravesoil, of very large cohorts of replicate mice (40 and 35, respectively).

### Substantial Findings of Epinecrotic Communities

Although many studies involving epinecrotic communities are largely exploratory, they are tremendously significant in making available a catalog of microbes that are present in and on decomposing remains. Dickson et al. (2011) published a revealing study of epinecrotic bacterial communities of detached swine heads immersed in an aquatic environment. The purpose of this study was to examine the potential of using sequencing data to estimate the length of time a body has been submerged in water. Three pig heads were completely immersed in Otago Harbor, New Zealand during autumn and winter. 16S PCR products were cloned and the bacterial sequences were identified. The result showed that marine bacteria rapidly colonized submerged remains in a successional manner. Bacteria detected on both autumn and winter carrion were primarily Proteobacteria. A significant finding was that time of year successional differences were observed, with progression occurring more rapidly in autumn than winter. In a recent related study, Benbow et al. (2015) used high-throughput 454-pyrosequencing to analyze bacterial communities of three stillborn piglet carcasses immersed in the Conestoga River in Pennsylvania, USA. The major finding was that winter and summer sampling identified Proteobacteria and Firmicutes as the most abundant phyla in both seasons, and their relative abundances were inversely related (Proteobacteria increased and Firmicutes decreased). Firmicutes are indigenous in decomposing bodies, but Proteobacteria originate from the environment and are ubiquitous in soil and water as the most abundant phylum on Earth (Roesch et al., 2007). Several studies have demonstrated that Proteobacteria and Firmicutes were the most ubiquitous and common bacteria in aquatic vertebrates (Han et al., 2010; Wu et al., 2010; Roeselers et al., 2011). Thus, it is probable that the increase in Proteobacteria in the Benbow et al. (2015) study is due to translocation of exogenous bacteria from aquatic environmental sources into the epinecrotic communities.

The exploratory study by Hyde et al. (2013) using 454-pyrosequencing demonstrated a marked shift in bacterial communities from aerobic to anaerobic bacteria in all tissues (mouth, zygotic arch, bicep, and torso) that were sampled from two cadavers placed at the Southeast Texas Applied Forensic Science Facility. The results produced three important findings: (i) there was variation in community signatures among cadavers; (ii) there was a difference between sample sites within a cadaver; and (iii) within a cadaver-sampling site, there was variation between bloat stage initial and end points.

Another informative study by Pechal et al. (2014) used 454-pyrosequencing to investigate the bacterial community composition of swabbed buccal cavities and skin of three swine carcasses decaying in a temperate forest in Ohio, USA. A proposed framework explained 94.4% of the time since placement of remains in the environment. Thus, this model provides an important prototype for estimating the number of days of placement by identifying bacterial communities. At the phyletic level, Proteobacteria and Firmicutes predominated; however, there was a substantial shift on the fifth day in which Firmicutes predominated on phyletic and familial levels. *Bacteroidaceae* and *Moraxellaceae* were significant family level indicators of the communities found on the first day of sampling, whereas *Bacillaceae* and *Clostridiales incertae sedis* XI were indicators of the 5 days postmortem.

Metcalf et al. (2013) endeavored to assess if peripheral epinecrotic succession was predictable through decomposition stages. The study employed rigorous sampling during a 48-day time-series study using replicate mice. Both 16S and 18S rRNA gene amplicons were sequenced. Sequence data associated with the bloat stage included Firmicutes (Lactobacillaceae and Bacteroidaceae families) that increased on the abdominal cavity. Proteobacteria was the dominant phylum throughout decomposition on the skin surfaces of both the head and torso areas. After carcasses ruptured, Firmicutes decreased drastically on the skin. Due to statistical significance and accuracy, a “microbial clock” was formulated, and PMI estimates corroborated actual time of death within 3 days. In general, a microbial clock is the time-dependent, postmortem synchronized succession of microbial diversity that occurs during the decaying process.

## Conclusion

This mini review has explored recent advances in microbiological studies involving the human postmortem microbiome. Death is an enigma; however, the study of microorganisms provides a missing piece of the postmortem microbial puzzle. The studies cited in this mini review reveal the tremendous progress in elucidating the identity and role of microbes in human putrefaction. There are a number of important questions that remain to be addressed concerning gene expression patterns of thanatomicrobiomic microbial diversity. RT-qPCR and microarrays are often used to monitor antemortem gene expression. A newly published report of apoptotic postmortem mRNA transcript abundance has been beneficial in providing molecular evidence of life after death (Javan et al., 2015). The study demonstrated that human mRNA can be extracted from liver tissues of actual criminal corpses, and that mRNA is informative for identifying postmortem gene expression patterns.

A theoretical framework that provides a prototype for studying the ecological successional patterns of thanatomicrobiomic and epinecrotic communities would be beneficial for microbiological studies of decomposition. Elucidating the trajectories and mechanisms regulating postmortem microbial succession is fundamental in hypothesizing the responses of microbes to postmortem change and forecasting their future conditions. Variation, structure, and function of microbial communities can be explained using abiotic (i.e., humidity and temperature) and biotic (i.e., gasses and insects) deterministic factors. These factors are limited and explain only 50% of the variation in microbial community structure (Zhou et al., 2013). Thus, variation in structure also includes stochastic processes. Microbial succession can vary between days to decades depending on variations in ecosystems, and perturbations. Putrefaction creates a nutrient-rich environment that generates perturbations to produce a domino effect through a microbial community that is hard to predict (Lemon et al., 2012). Therefore, stochastic processes play a less important role before perturbations caused by death as compared to deterministic processes.

### Future Directions

Two under-developed areas of thanatomicrobiomic and epinecrotic studies are the use of the nuclear ribosomal internal transcribed spacer (ITS) to characterized fungi, and the use of archaea because the abundance are generally too low (Lauber et al., 2014; Metcalf et al., 2016). Future studies should address standardization of postmortem tissue collection, DNA extraction protocols, and sequencing platforms. For example, researchers with the HMP Consortium developed agreed-upon protocols to characterize DNA in 5,177 microbial taxonomic profiles from their collection of 11,174 biological specimens (The Human Microbiome Project Consortium, 2012).A new project, the human postmortem microbiome project (HPMP), could presumably lead to the cultivation of forensic and investigative tools and databases for use by medicolegal and forensic researchers. This project would study the role of microorganisms in PMI and cause of death determinations. Similar collaborative methods such as the swabbing of tissue aliquots versus tissue dissection, uniform extraction methods, and standardized sequencing platforms similar to what is suggested by the Earth Microbiome Project (Gilbert et al., 2010) and HMP can be implemented. Through concerted efforts to refine, verify, and standardize protocols, a framework for essential tools for thanatomicrobiomic and epinecrotic investigations is imminent and will advance the understanding of the role of microbes in human putrefaction.

## Author Contributions

GJ, SF, ZA, and JM contributed equally to the conception, drafting, and editing of this mini-review.

## Conflict of Interest Statement

The authors declare that the research was conducted in the absence of any commercial or financial relationships that could be construed as a potential conflict of interest.

## References

[B1] Benbow M. E. Pechal J. L. Lang J. M. Erb R. Wallace J. R. (2015). The potential of high-throughput metagenomic sequencing of aquatic bacterial communities to estimate the postmortem submersion interval. *J. Forensic Sci.* 60 1500–1510. 10.1111/1556-4029.12859 26294275

[B2] Benninger L. A. Carter D. O. Forbes S. L. (2008). The biochemical alteration of soil beneath a decomposing carcass. *Forensic Sci. Int.* 180 70–75. 10.1016/j.forsciint.2008.07.001 18752909

[B3] Campobasso C. P. Di Vella G. Introna F. (2001). Factors affecting decomposition and Diptera colonization. *Forensic Sci. Int.* 120 18–27. 10.1016/S0379-0738(01)00411-X 11457604

[B4] Can I. Javan G. T. Pozhitkov A. E. Noble P. A. (2014). Distinctive thanatomicrobiome signatures found in the blood and internal organs of humans. *J. Microbiol. Methods* 106 1–7. 10.1016/j.mimet.2014.07.026 25091187

[B5] Caporaso J. G. Lauber C. L. Walters W. A. Berg-Lyons D. Lozupone C. A. Turnbaugh P. J. (2011). Global patterns of 16S rRNA diversity at a depth of millions of sequences per sample. *Proc. Natl. Acad. Sci. U.S.A.* 108(Suppl. 1), 4516–4522. 10.1073/pnas.1000080107 20534432 PMC3063599

[B6] Carter D. O. Metcalf J. L. Bibat A. Knight R. (2015). Seasonal variation of postmortem microbial communities. *Forensic Sci. Med. Pathol.* 11 202–207. 10.1007/s12024-015-9667-7 25737335 PMC9636889

[B7] Carter D. O. Yellowlees D. Tibbett M. (2008). Temperature affects microbial decomposition of cadavers (*Rattus rattus*) in contrasting soils. *Appl. Soil Ecol.* 40 129–137. 10.1016/j.apsoil.2008.03.010

[B8] Carter D. O. Yellowlees D. Tibbett M. (2010). Moisture can be the dominant environmental parameter governing cadaver decomposition in soil. *Forensic Sci. Int.* 200 60–66. 10.1016/j.forsciint.2010.03.031 20400249

[B9] Chun L. P. Miguel M. J. Junkins E. N. Forbes S. L. Carter D. O. (2015). An initial investigation into the ecology of culturable aerobic postmortem bacteria. *Sci. Justice* 55 394–401. 10.1016/j.scijus.2015.07.003 26654073

[B10] Damann F. E. Williams D. E. Layton A. C. (2015). Potential use of bacterial community succession in decaying human bone for estimating postmortem interval. *J. Forensic Sci.* 60 844–850. 10.1111/1556-4029.12744 25808627

[B11] Dickson G. C. Poulter R. T. Maas E. W. Probert P. K. Kieser J. A. (2011). Marine bacterial succession as a potential indicator of postmortem submersion interval. *Forensic Sci. Int.* 209 1–10. 10.1016/j.forsciint.2010.10.016 21074955

[B12] Fierer N. Lauber C. L. Zhou N. McDonald D. Costello E. K. Knight R. (2010). Forensic identification using skin bacterial communities. *Proc. Natl. Acad. Sci. U.S.A.* 107 6477–6481. 10.1073/pnas.1000162107 20231444 PMC2852011

[B13] Ford W. W. (1901). On the bacteriology of normal organs. *J. Hyg. (Lond.)* 1 277–284. 10.1017/S0022172400000231 20474121 PMC2235945

[B14] Fredette J. W. (1916). Bacteremias in the agonal period. *J. Lab. Clin. Med.* 2 180–188.

[B15] Gilbert J. A. Meyer F. Antonopoulos D. Balaji P. Brown C. T. Brown C. T. (2010). Meeting report: the terabase metagenomics workshop and the vision of an Earth microbiome project. *Stand. Genomic Sci.* 3 243–248. 10.4056/sigs.1433550 21304727 PMC3035311

[B16] Hamady M. Knight R. (2009). Microbial community profiling for human microbiome projects: tools, techniques, and challenges. *Genome Res.* 19 1141–1152. 10.1101/gr.085464.108 19383763 PMC3776646

[B17] Han S. Liu Y. Zhou Z. He S. Cao Y. Shi P. (2010). Analysis of bacterial diversity in the intestine of grass carp (*Ctenopharyngodon idellus*) based on 16S rDNA gene sequences. *Aquac. Res.* 42 47–56. 10.1111/j.1365-2109.2010.02543.x

[B18] Hauther K. A. Cobaugh K. L. Jantz L. M. Sparer T. E. DeBruyn J. M. (2015). Estimating time since death from postmortem human gut microbial communities. *J. Forensic Sci.* 60 1234–1240. 10.1111/1556-4029.12828 26096156

[B19] Heimesaat M. M. Boelke S. Fischer A. Haag L. M. Loddenkemper C. Kühl A. A. (2012). Comprehensive postmortem analyses of intestinal microbiota changes and bacterial translocation in human flora associated mice. *PLoS ONE* 7:e40758 10.1371/journal.pone.0040758 PMC3395637 22808253

[B20] Howard G. T. Duos B. Watson-Horzelski E. J. (2010). Characterization of the soil microbial community associated with the decomposition of a swine carcass. *Int. Biodeterior. Biodegradation* 64 300–304. 10.1016/j.ibiod.2010.02.006

[B21] Hyde E. R. Haarmann D. P. Lynne A. M. Bucheli S. R. Petrosino J. F. (2013). The living dead: bacterial community structure of a cadaver at the onset and end of the bloat stage of decomposition. *PLoS ONE* 8:e77733 10.1371/journal.pone.0077733 PMC3813760 24204941

[B22] Hyde E. R. Haarmann D. P. Petrosino J. F. Lynne A. M. Bucheli S. R. (2015). Initial insights into bacterial succession during human decomposition. *Int. J. Legal Med.* 129 661–671. 10.1007/s00414-014-1128-4 25431049

[B23] Javan G. T. Can I. Finley S. J. Soni S. (2015). The apoptotic thanatotranscriptome associated with the liver of cadavers. *Forensic Sci. Med. Pathol.* 11 509–516. 10.1007/s12024-015-9704-6 26318598

[B24] Lauber C. L. Metcalf J. L. Keepers K. Ackermann G. Carter D. O. Knight R. (2014). Vertebrate decomposition is accelerated by soil microbes. *Appl. Environ. Microbiol.* 80 4920–4929. 10.1128/AEM.00957-14 24907317 PMC4135757

[B25] Lemon K. P. Armitage G. C. Relman D. A. Fischbach M. A. (2012). Microbiota-targeted therapies: an ecological perspective. *Sci. Transl. Med.* 4 137rv5. 10.1126/scitranslmed.3004183 PMC5725196 22674555

[B26] Metcalf J. L. Parfrey L. W. Gonzalez A. Lauber C. L. Knights D. Ackermann G. (2013). A microbial clock provides an accurate estimate of the postmortem interval in a mouse model system. *Elife* 2 e01104 10.7554/eLife.01104 PMC3796315 24137541

[B27] Metcalf J. L. Xu Z. Z. Weiss S. Lax S. Van Treuren W. Hyde E. R. (2016). Microbial community assembly and metabolic function during mammalian corpse decomposition. *Science* 351 158–162. 10.1126/science.aad2646 26657285

[B28] Morris J. A. Harrison L. M. Partridge S. M. (2006). Postmortem bacteriology: a re-evaluation. *J. Clin. Pathol.* 59 1–9. 10.1136/jcp.2005.028183 16394274 PMC1860254

[B29] Pechal J. L. Crippen T. L. Benbow M. E. Tarone A. M. Dowd S. Tomberlin J. K. (2014). The potential use of bacterial community succession in forensics as described by high throughput metagenomic sequencing. *Int. J. Legal Med.* 128 193–205. 10.1007/s00414-013-0872-1 23749255

[B30] Pechal J. L. Crippen T. L. Tarone A. M. Lewis A. J. Tomberlin J. K. Benbow M. E. (2013). Microbial community functional change during vertebrate carrion decomposition. *PLoS ONE* 8:e79035 10.1371/journal.pone.0079035 PMC3827085 24265741

[B31] Roesch L. F. Fulthorpe R. R. Riva A. Casella G. Hadwin A. K. Kent A. D. (2007). Pyrosequencing enumerates and contrasts soil microbial diversity. *ISME J.* 1 283–290. 10.1038/ismej.2007.53 18043639 PMC2970868

[B32] Roeselers G. Mittge E. K. Stephens W. Z. Parichy D. M. Cavanaugh C. M. Guillemin K. (2011). Evidence for a core gut microbiota in the zebrafish. *ISME J.* 5 1595–1608. 10.1038/ismej.2011.38 21472014 PMC3176511

[B33] Sender R. Fuchs S. Milo R. (2016). Are we really vastly outnumbered? Revisiting the ratio of bacterial to host cells in humans. *Cell* 164 337–340. 10.1016/j.cell.2016.01.013 26824647

[B34] Sibulesky L. (2013). Normal liver anatomy. *Clin. Liver Dis.* 2 S1–S3. 10.1002/cld.124 PMC6448666 30992874

[B35] Stewart E. J. (2012). Growing unculturable bacteria. *J. Bacteriol.* 194 4151–4160. 10.1128/JB.00345-12 22661685 PMC3416243

[B36] The Human Microbiome Project Consortium (2012). Structure, function and diversity of the healthy human microbiome. *Nature* 486 207–214. 10.1038/nature11234 22699609 PMC3564958

[B37] Tuomisto S. Karhunen P. J. Vuento R. Aittoniemi J. Pessi T. (2013). Evaluation of postmortem bacterial migration using culturing and real-time quantitative PCR. *J. Forensic Sci.* 58 910–916. 10.1111/1556-4029.12124 23550887

[B38] Tuomisto S. Pessi T. Collin P. Vuento R. Aittoniemi J. Karhunen P. J. (2014). Changes in gut bacterial populations and their translocation into liver and ascites in alcoholic liver cirrhotics. *BMC Gastroenterol.* 14:40 10.1186/1471-230X-14-40 PMC3996058 24564202

[B39] Turnbaugh P. J. Ley R. E. Hamady M. Fraser-Liggett C. Knight R. Gordon J. I. (2007). The human microbiome project: exploring the microbial part of ourselves in a changing world. *Nature* 449 804–810. 10.1038/nature06244 17943116 PMC3709439

[B40] Williams K. P. Gillespie J. J. Sobral B. W. Nordberg E. K. Snyder E. E. Shallom J. M. (2010). Phylogeny of gammaproteobacteria. *J. Bacteriol.* 192 2305–2314. 10.1128/JB.01480-09 20207755 PMC2863478

[B41] Wu S. Gao T. Zheng Y. Wang W. Cheng Y. Wang G. (2010). Microbial diversity of intestinal contents and mucus in yellow catfish (*Pelteobagrus fulvidraco*). *Aquaculture* 30 1–7. 10.1016/j.aquaculture.2009.12.025

[B42] Zhou J. Liu W. Deng Y. Jiang Y. H. Xue K. He Z. (2013). Stochastic assembly leads to alternative communities with distinct functions in a bioreactor microbial community. *MBio* 4 e00584-12 10.1128/mBio.00584-12 PMC3585448 23462114

